# Atypical Colonoscopic Presentation of Lymphocytic Colitis Mimicking Hyperplastic Polyposis Syndrome

**DOI:** 10.7759/cureus.2159

**Published:** 2018-02-05

**Authors:** Umair Iqbal, Hafsa Anwar, Abdulhadi A Quadri

**Affiliations:** 1 Internal Medicine, Bassett Medical Center; 2 Jinnah Sindh Medical University, Dow University of Health Sciences (DUHS), Karachi, Pakistan; 3 Gastroenterology, Bassett Medical Center

**Keywords:** lymphocytic colitis, microscopic colitis, hyperplastic polyposis syndrome, chronic diarrhea

## Abstract

Lymphocytic colitis is a chronic inflammatory disease of colon usually presented in middle age female as chronic watery diarrhea. Diagnosis is made on biopsy as colonoscopy usually revealed normal appearing colonic mucosa. We present here an unusual case of a 25-year-old female with past medical history of asthma was evaluated for one year of non-bloody watery diarrhea. The symptoms started after a course of antibiotics for upper respiratory tract infection a year back. The serum chemistries, including liver enzymes, were unremarkable. Stool culture, ova, and parasites were unremarkable. Stool Clostridium difficile was also negative. Celiac disease antibodies were unremarkable. Stool occult blood test was positive. The patient underwent colonoscopy for the evaluation of chronic diarrhea and revealed multiple polyps throughout the colon with inflamed surfaces which were biopsied. The concern was for hyperplastic polyposis syndrome. Genetic testing for adenomatous polyposis gene was done and came back negative. Biopsy from polyps revealed lymphocytic colitis. The patient was started on budesonide which resulted in marked improvement in her symptoms. Our case highlighted an atypical endoscopic finding of lymphocytic colitis which mimic hyperplastic polyposis syndrome.

## Introduction

Microscopic colitis is a chronic inflammatory disease of the colon that usually presents with chronic, non-bloody, and watery diarrhea [1]. It has two well-defined types: collagenous colitis and lymphocytic colitis. Collagenous colitis is characterized by the presence of colonic intraepithelial collagen bands greater than 10 mm, and lymphocytic colitis is diagnosed due to the presence of colonic intraepithelial lymphocytic infiltrate [2-3]. The diagnosis of microscopic colitis and differentiation of its types is usually done after biopsy as clinical signs and symptoms are similar in both types. The colonoscopy usually reveals normal-appearing colonic mucosa or shows non-specific changes. We present here an unusual case of lymphocytic colitis mimicking hyperplastic polyposis syndrome.

## Case presentation

**Figure 1:**
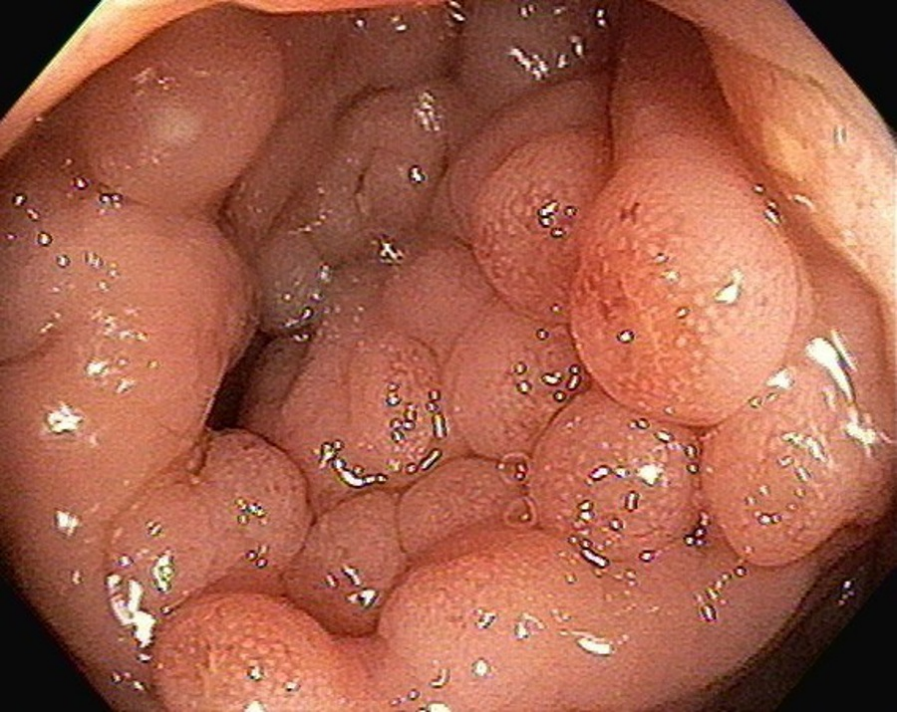
Multiple polyps seen throughout the colon

A 25-year-old female with past medical history of irritable bowel syndrome and asthma was evaluated for one year of chronic watery diarrhea. She reported three to four watery bowel movements daily, which were associated with abdominal cramping. No blood reported in the stool. No changes in weight or appetite were reported. She denied any fever or chills. She reported that symptoms started after a course of antibiotics for an upper respiratory tract infection that occurred the previous year. She denied any recent travel or use of any herbal supplements. No medication use reported. The physical exam, including vital signs on presentation, was unremarkable. The serum chemistries, including liver enzymes, were unremarkable. Stool culture, ova, and parasites were unremarkable. Stool Clostridium difficile was also negative. Celiac disease antibodies were unremarkable. Stool occult blood test was positive. She was screened for Celiac disease and inflammatory bowel diseases but results were negative for tissues transglutaminase, anti-endomysial, and saccharomyces cerevisiae antibodies. The patient underwent colonoscopy for the evaluation of chronic diarrhea and revealed multiple polyps throughout the colon with inflamed surfaces which were biopsied (Figure 1).

**Figure 2:**
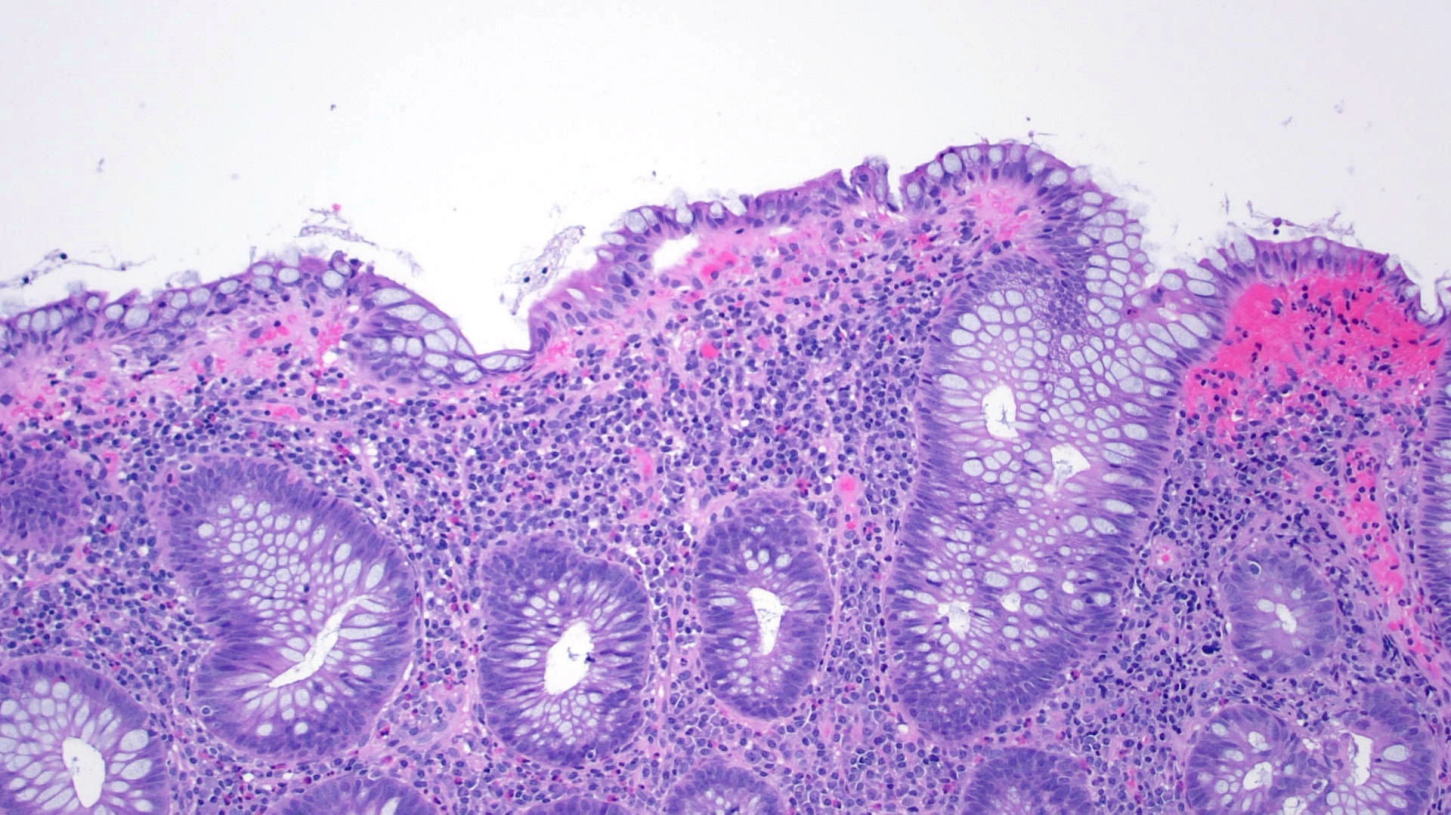
Biopsy revealed increased lymphoplasmacellular inflammatory cells within an expanded lamina propria, with mild increase in sub-epithelial collagen, and increased amounts of intraepithelial lymphocytes

Biopsy from polyps revealed chronic colitis with features of lymphocytic colitis. The lamina propria shows dense plasma cells and lymphocytic infiltration mixed with eosinophils and neutrophils (Figure 2).

The concern was for hyperplastic polyposis syndrome given the morphological appearance of diffuse polyposis seen on colonoscopy. Genetic testing for adenomatous polyposis gene was done and came back negative. The patient was started on tapering doses of budesonide with improvement in her symptoms. 

## Discussion

The estimated incidence of lymphocytic colitis is about 3 per 100,000 [4]. It usually presents in middle-aged and elderly females but cases have reported it occurring in young patients and children [4-5]. Chronic non-bloody diarrhea with or without abdominal pain is a very common presentation though some patients may present with irritable bowel disease-like symptoms (diarrhea alternating with constipation), or rarely with constipation [1, 6]. Abdominal pain can be present in 50% of the patients. Extra-intestinal symptoms like arthralgia, arthritis, and uveitis can be present and may warrant screening for associated conditions. Smoking and use of non-steroidal anti-inflammatory drugs (NSAIDs) are associated with increased risk of developing this disease [7]. It is found to be associated with multiple autoimmune diseases including Celiac disease, autoimmune thyroiditis, Type 1 diabetes mellitus, and non-erosive, oligoarticular arthritis [8]. Laboratory findings are usually non-specific, with mild elevation of erythrocyte sedimentation rate (ESR) and the presence of autoantibodies in 50% of patients. Diagnoses are established with a colonoscopy along with a mucosal biopsy, and additionally, the severity of diarrhea usually correlates with the inflammatory changes seen in the lamina propria. The colonoscopy usually reveals normal-appearing mucosa or shows non-specific changes of erythema; friability may be seen [9]. Our patient had an atypical endoscopic finding with the presence of diffuse polyposis throughout the colon which is usually seen in hyperplastic polyposis syndrome, a rare condition characterized by multiple, large and/or proximal serrated polyps. A study done on 795 patients with microscopic colitis revealed the presence of colorectal polyps in only 5% of the patients but diffuse polyposis was not reported in any of the patients [9].

Cronkhite-Canada syndrome (CCS) is also another type of diffuse polyposis syndrome which presents with chronic bloody or non-bloody diarrhea, usually affecting Asians, and is associated with alopecia and loss of finger and toenails. The diagnosis is made following clinical presentation and biopsy. Our patient did not have any nail changes or hair loss that were concerning for CCS and biopsy eventually ruled out CCS and revealed lymphocytic colitis. The primary goal in the treatment of patients with lymphocytic colitis is to achieve control of the diarrheal symptoms. Patients with the active disease can benefit from budesonide therapy, as several randomized control trials have shown its efficacy in achieving remission [10]. Diarrhea may resolve within weeks of treatment but recurrence of symptoms are common. Approximately 10% to 20% of patients are refractory to budesonide treatment. Combination of cholestyramine and loperamide or bismuth subsalicylate can be considered in patients not responding to initial treatment with budesonide. Smoking cessation and avoidance of NSAIDs should be recommended in all patients.

## Conclusions

Lymphocytic colitis is characterized by chronic non-bloody diarrhea in young to middle-aged females. The diagnosis is usually made on biopsy as colonoscopy usually reveals normal-appearing colonic mucosa. Our case highlighted an atypical endoscopic finding of this entity which mimics hyperplastic polyposis syndrome through the presence of diffuse polyposis throughout the colon.
